# The complex challenge of governing food systems: The case of South African food policy

**DOI:** 10.1007/s12571-022-01258-z

**Published:** 2022-02-25

**Authors:** Sandra Boatemaa Kushitor, Scott Drimie, Rashieda Davids, Casey Delport, Corinna Hawkes, Tafadzwanashe Mabhaudhi, Mjabuliseni Ngidi, Rob Slotow, Laura M. Pereira

**Affiliations:** 1 Food Security Initiative, Stellenbosch University, Stellenbosch, South Africa; 2 Centre for Sustainability Studies, Stellenbosch University, Stellenbosch, South Africa; 3 Sustainable and Healthy Food Systems (SHEFS). School of Agricultural, Earth and Environmental Sciences, University of KwaZulu-Natal, Private Bag X01, Scottsville 3209, Pietermaritzburg, South Africa; 4 Department of Agricultural Economics, Stellenbosch University, Stellenbosch, South Africa; 5 Centre for Food Policy, City, University of London, London, United Kingdom; 6 Centre for Transformative Agricultural and Food Systems, School of Agricultural, Earth and Environmental Sciences, University of KwaZulu-Natal, Private Bag X01, Scottsville 3209, Pietermaritzburg, South Africa; 7 Department of Agricultural Extension and Rural Resource Management, School of Agriculture, Earth and Environmental Sciences, University of KwaZulu-Natal, Private Bag X01, Scottsville 3209, Pietermaritzburg, South Africa _Ste_; 8 School of Life Sciences, University of KwaZulu-Natal, Private Bag X01, Scottsville 3209, Pietermaritzburg, South Africa; 9 Department of Genetics, Evolution, and Environment, University College, London

**Keywords:** Food policy, Food systems, Sustainable food systems, Food security, Nutrition, South Africa

## Abstract

International experience reveals that food policy development often occurs in silos and offers few tangible mechanisms to address the interlinked, systemic issues underpinning food and nutrition insecurity. This paper investigated what South African government policies cover in terms of different aspects of the food system, who is responsible for them, and how coordinated they are. Policy objectives were categorized into seven policy domains relevant to food systems: agriculture, environment, social protection, health, land, education, economic development, and rural development. Of the ninety-one policies reviewed from 1947-2017, six were identified as being “overarching” with goals across all the domains. About half of the policies focused on agriculture and the environment, reflecting an emphasis on agricultural production. Policies were formulated and implemented in silos. As a result, learning from implementation, and adjusting to improve impact has been limited. Particularly important is that coordination during implementation, across these complex domains, has been partial. In order to achieve its stated food and nutrition outcomes, including Sustainable Development Goal (SDG) 2, South Africa needs to translate its policies into tangible, practical plans and processes guided by effective coordination and alignment. Key recommendations are practically to align policies to a higher-level “food goal”, establish better coordination mechanisms, consolidate an effective monitoring and evaluation approach to address data gaps and encourage learning for adaptive implementation. Actively engaging the existing commitments to the SDGs would draw stated international commitments together to meet the constitutional commitment to food rights into an overarching food and nutrition security law.

## Introduction

The Sustainable Development Goals (SDGs) reflect a growing global consensus on the need to address sustainability challenges (United Nations 2015). SDG 2 aims to end hunger, achieve food security, improve nutrition, and promote sustainable agriculture. This outcome requires change across multiple domains, including health, development, and agriculture. Achieving this goal requires transformative change across the food system (Willett et al. 2019) and the political will to effect such changes to recognize the integrated nature of food systems (Candel and Pereira 2017). In essence, food governance has to underpin the ability of present and future generations to meet their food and nutrition needs under extraordinary environmental pressures (Gordon et al. 2017; Springmann et al. 2018).

Despite sufficient available food, hunger persists alongside growing rates of overweight and obesity (Ng et al. 2014). This has raised questions about the current modes of food production, consumption, and food system governance, particularly as environmental impacts have become stark (Campbell et al. 2017; Gill et al. 2015; Gordon et al. 2017; Tilman and Clark 2014; Willett et al. 2019). Policy responses have not met the scale of the challenge (Mason and Lang 2017). Responses across governments have often been fragmented, with competing interests playing out over food security and agricultural production, versus consumption practices and health challenges (Lang and Heasman 2015). Failing food systems have impacts beyond health, contributing to global environmental change (Gill et al. 2015; Tilman and Clark 2014), impeding economic growth (Global Panel 2017), and exacerbating socio-economic inequalities (Hawkes 2006). Given these global trends, policy that can enable more sustainable and healthy food systems remain a critical challenge.

Food system governance has suffered from policy disintegration during formulation and implementation (Balarajan and Reich 2016; Harris et al. 2017; Hendriks 2014; Hendriks et al. 2017a; Kiguli et al. 2019; Peters 2018; Termeer et al. 2018). Food systems have been defined as activities ranging from production to consumption (Ericksen 2008; HLPE 2020). The production and consumption of food bring together biophysical and social elements encompassing multiple subsystems that require action from different sectors. Coordinating the actors, their interests, and activities of all these diverse subsystems in ways that produce synergy has been a challenge (Poole et al. 2021; Walls et al. 2019). Actions from and across all these various subsystems have been impeded by stakeholder interests, specialisation, power, political trade-offs, and accountability, among others (Baker et al. 2018; Committee on Food Security 2018; Peters 2018). These coordination failures have hindered the possibility of change in the food system despite multiple attempts and emphasise the need for rethinking and reforming global coordination food systems (HLPE 2020).

As a strong constitutional democracy, South Africa provides a critical case study for other low-and-middle-income countries to understand how these challenges, including meeting SDG2, are being met through policy responses. Despite having a plethora of food system policies (Supplementary File 1), the country is failing to address the food needs of its citizens (Boatemaa et al. 2018; Hendriks 2018). This study seeks to understand the complex challenge of governing food systems by interrogating the country’s policy response to the environmental, health, and socio-economic aspects of food from a political economy perspective. By focusing on the policy component of governance, we establish what policies exist, their focus in the food system, under what parts of government they fall, and the coordination during policy implementation. The purpose is twofold. First, to map the existing policy landscape relevant to food systems in South Africa. Second, to draw broader lessons about how food could be more effectively governed to address challenges and achieve the SDGs. We conducted a systemic review of national food system policies to: 1) identify and understand policies as one of the key drivers of food system change and 2) identify the coherence among food system-related policies.

### The case of South Africa

The South African food system is highly contested, with the Apartheid legacy underpinning a dualistic agrarian system, high levels of poverty, and social-economic inequality (Aliber and Hall 2012; S Greenberg 2017; Stephen Greenberg 2010). South Africa has a high per capita income for a developing country and is food secure at the national level (Scott Drimie and Ruysenaar 2010; McLaren et al. 2015). However, the country faces a higher burden of malnutrition than countries of comparable income levels (Statistics South Africa 2016) and is undergoing nutrition and epidemiological transition (Steyn and Mchiza 2014; Tathiah et al. 2013). Social, economic, and ecological factors lead to between 23 and 30% of the population having severe inadequate access to food or being at risk of hunger (Ledger 2016; Statistics South Africa 2016).

Food security can be understood as the state when all people, at all times, have physical and economic access to sufficient, safe, and nutritious food that meets their dietary needs and food preferences for an active and healthy life (FAO 1996). In South Africa, food security mainly depends on income rather than agricultural production, even in rural areas (Pereira et al. 2014). However, buying a nutritionally acceptable diet is beyond the financial ability of many households (Faber and Drimie 2016; Schönfeldt et al. 2013). According to the National Agricultural Marketing Council, a nutritious food basket was valued at USD 40^1^ in May 2016 (National Agricultural Marketing Council 2016). This represented 35-40% of total income earned in low-income households. Poor households spend 8% of their income on vegetables, while rich households spend only 1% (Jansen et al. 2012). With other pressing needs such as shelter, water, electricity, and transport, many cannot afford to spend 40% of their wages on food.

Since the transition to democratic governance in 1994, the government has developed policies to address the structural factors that have sustained hunger and overnutrition (McLaren et al. 2015). These initiatives are primarily informed by Section 27 of the Constitution, which guarantees that *‘everyone has the right to access sufficient food and water’* (Republic of South Africa 1996). These policy interventions include supporting land reform, social protection programmes, field crop production, nutrition education, the school nutrition programme, and lowering the price of bread and some fruits and vegetables (Boatemaa et al. 2018). After years of implementation, these policies have demonstrated some impacts on improving stunting, but not over-nutrition, micronutrient deficiency and environmental security (Dugard 2015; S. Hendriks 2013; Pereira and Drimie 2016). Overall, most government initiatives emphasise agricultural productivity, and not the root causes of food insecurity, including structural poverty, inequality, and environmental degradation (Klerk et al. 2004; Termeer et al. 2018).

## Materials and methods

A political economy lens guides this research. Political economy is defined as understanding the effects of political and economic decisions on development interventions (Food and Agriculture Organisation 2017). A political economy analysis of food systems typically includes stakeholder analysis, their relations, institutions, process ideas, and their influence over policy formulation and implementation (Gillespie et al. 2013; Leach et al. 2020; Nisbett et al. 2014). This study analysed the government institutions involved in food system policies, the domains covered by policies, and policy coordination. We conducted a systemic review of national policies related to the food system, which had been gazetted before March 2017. We defined policies as decisions of government that are codified in the Constitution, Acts, white papers, green papers, regulations, norms and standards, strategies, plans, and policies (Birkland, 2010). Through government websites and a Google search, we identified sixty-nine policies related to producing/rearing/catching/foraging, processing, packaging, distributing and retailing, and consuming food (Ericksen 2008). The list was updated after comparison with academic papers by Hendriks et al. (2017), Boatemaa et al. (2018), and Drimie (2016). A comprehensive database of 97 policies was created after a review by the SHEFS (Sustainable and Healthy Food Systems) research team and government officials. Data from 91 of the policies are presented in this paper after six policies were omitted as they were in draft form.

We adopted the approach developed by Harris and colleagues to categorise the policies into seven domains (Agriculture, Environment, Social Protection, Health, Land, Education, and Rural Development) under which the policies could be clustered (Harris et al. 2017).

Keywords were defined for each of the domains. The keywords were developed based on food system activities and outcomes related to sustainability. Policy documents were scanned for these keywords. After the keywords search, the text retrieved was assessed to determine the context in which each keyword was used. A text was considered as background, axis, objective, or strategy. Background text referred to an introduction, context, or background for the policy. Axis was the text that described the relevant problem for which the policy was designed to address. The objective was the text that stated the aims, objectives, vision, goals, and plans of the policy. Strategies were the text that explained the different strategies in the policy for achieving the objective. These included actions, participating stakeholders, and coordination mechanisms, including interdependence among sectors.

Policy coordination is defined as information, resources, and responsibility sharing to achieve a specific outcome. Effective coordination of food system policies requires a shared understanding for cross-sectoral activities, concrete institutional arrangements, and an active learning and adapting of policy and programming to ensure impact (Drimie et al. 2014). Policy coordination was measured with three pieces of evidence 1) how different sectoral policies articulated interdependence with other sectors, 2) whether mechanisms to enable coordination between sectoral policies and programmes were defined, 3) and whether learning and adapting (a “learning ethos”) from implementation was established to enable policies to adapt to a fast-changing context (Table 1). Each of these questions was used as a code, and we looked for the absence or presence of these codes in the policies. For example, for the interdependence amongst sectors, each domain’s main strategy was assessed for whether links or connections with other government and non-state actors were defined.

The analysis team consisted of four authors (SBK, CD, SD, and LP). A team meeting was held to draw up the coding frame after all team members read the first ten policies. The remaining documents were simultaneously coded by two authors (SBK and CD). When uncertain about a code for a particular statement, this was referred to SD and LP for inputs. A code was selected after discussion and consensus of at least three team members.

## Results

### Departments involved in policymaking

This study included ninety-one policies. The highest proportion of policies was made by the Department of Agriculture, Forestry, and Fisheries (DAFF) (34%), followed by the Department of Health (DoH) (15%) and the Department of Environmental Affairs (DEA) (17%) (Supplementary File 1 contains the list of abbreviations). As a result of the 2019 cabinet reshuffle, some departments have been reconstituted with new names, but the previous departmental names are kept for this study (Supplementary File 1).

### Timeline of policies

**Figure 1:**
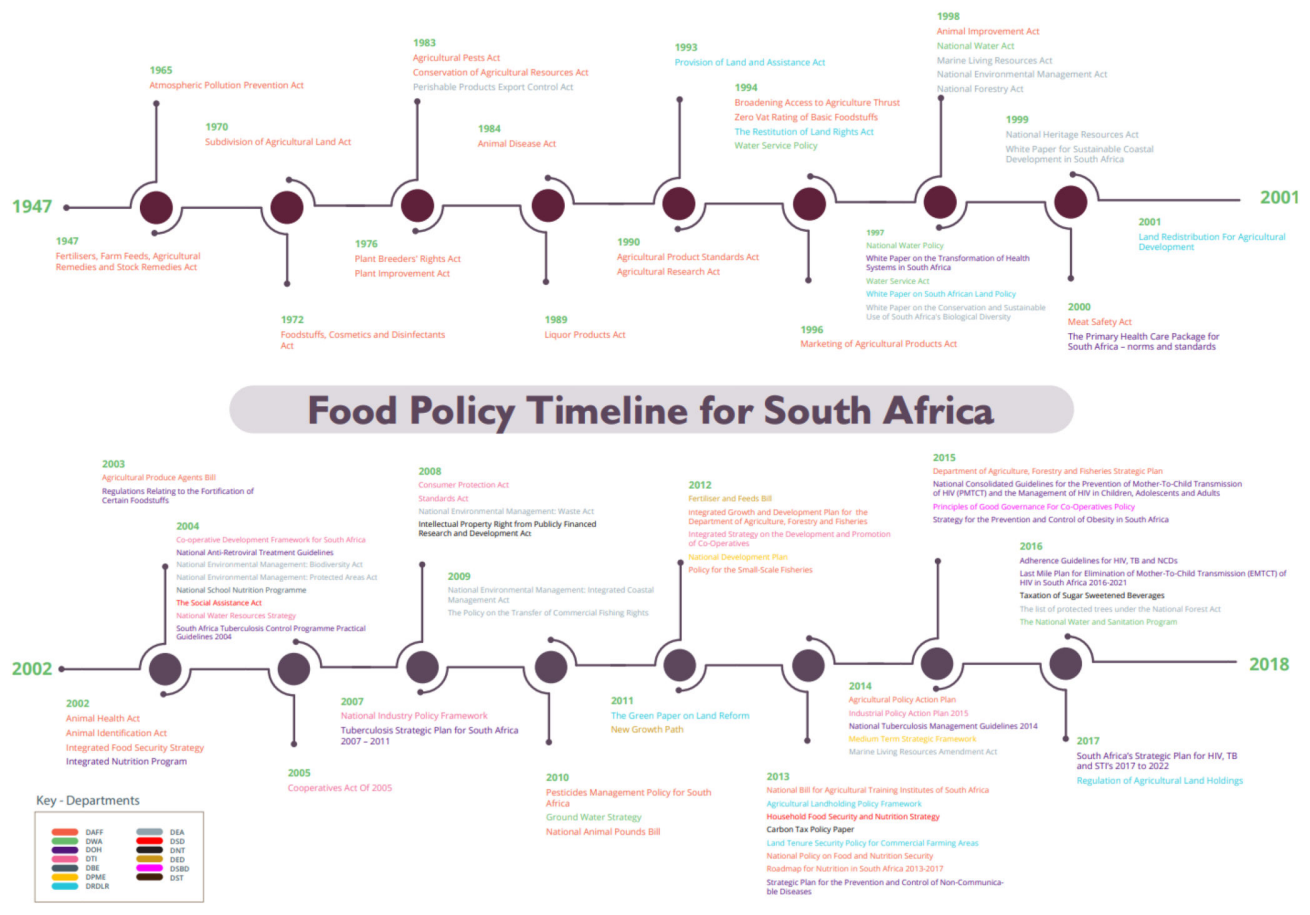
Timeline of extant food system-related policies in South Africa from 1947 – 2017

The first policy was enacted in 1947; the Fertilisers, Farm Feeds, Agricultural Remedies, and Stock Remedies Act (Figure 1). Extant policies that followed this until 1994 focused mainly on conservation, land, agricultural production, and national food security. Food security was overwhelmingly conceptualised as an agricultural production issue. It was a key priority of Apartheid-era policymakers, including the Plant Improvement Act, the Agricultural Pests Act, and the Agricultural Research Act.

In line with global debates about food security, the post-1994 policies moved beyond food production towards improvement in livelihoods, with an increasing focus on household and individual nutrition security. Underpinned by the South African Constitution, key policies included Zero-Rated Vat on some food items, the Primary School Nutrition Programme (revamped into the National School Nutrition Program), the Integrated Food Security Strategy (IFSS), Integrated Nutrition Program (INP), the Social Assistance Act, and the Social Relief of Distress Grants (Chagunda 2014; Department of Social Development 2002; Jansen et al. 2012; Rendall-Mkosi et al. 2013; Republic of South Africa 1996). In addition, other policies focused on the redistribution of productive assets, especially land.

### Food system domains covered by the policies

In 2010, the National Planning Commission (NPC) reported that South Africa was slow to progress on improving food security due to a general failure to implement policies and an absence of broad partnerships (Hendriks 2013). The National Development Plan (NDP) was developed to address this problem by 2030 (National Planning Commission 2011). It is the overarching government development plan across all levels of government. The New Growth Path (NGP), the Medium-Term Strategic Framework (MTSF) 2014-2019, and the Industrial Policy Action Plan (IPAP) were developed to support the NDP (Nattrass 2011). The IFSS reflected the first broad, interdepartmental initiative on food security (DAFF 2012). The Roadmap for Nutrition in South Africa (2013-2017) (RNSA) and the DAFF Strategic Framework 2015-2020 are also important strategic documents (Department of Health 2013). In 2013, the National Policy on Food and Nutrition Security (NPFNS) was gazetted (Department of Agriculture Forestry and Fisheries (DAFF) 2013).

**Figure 2:**
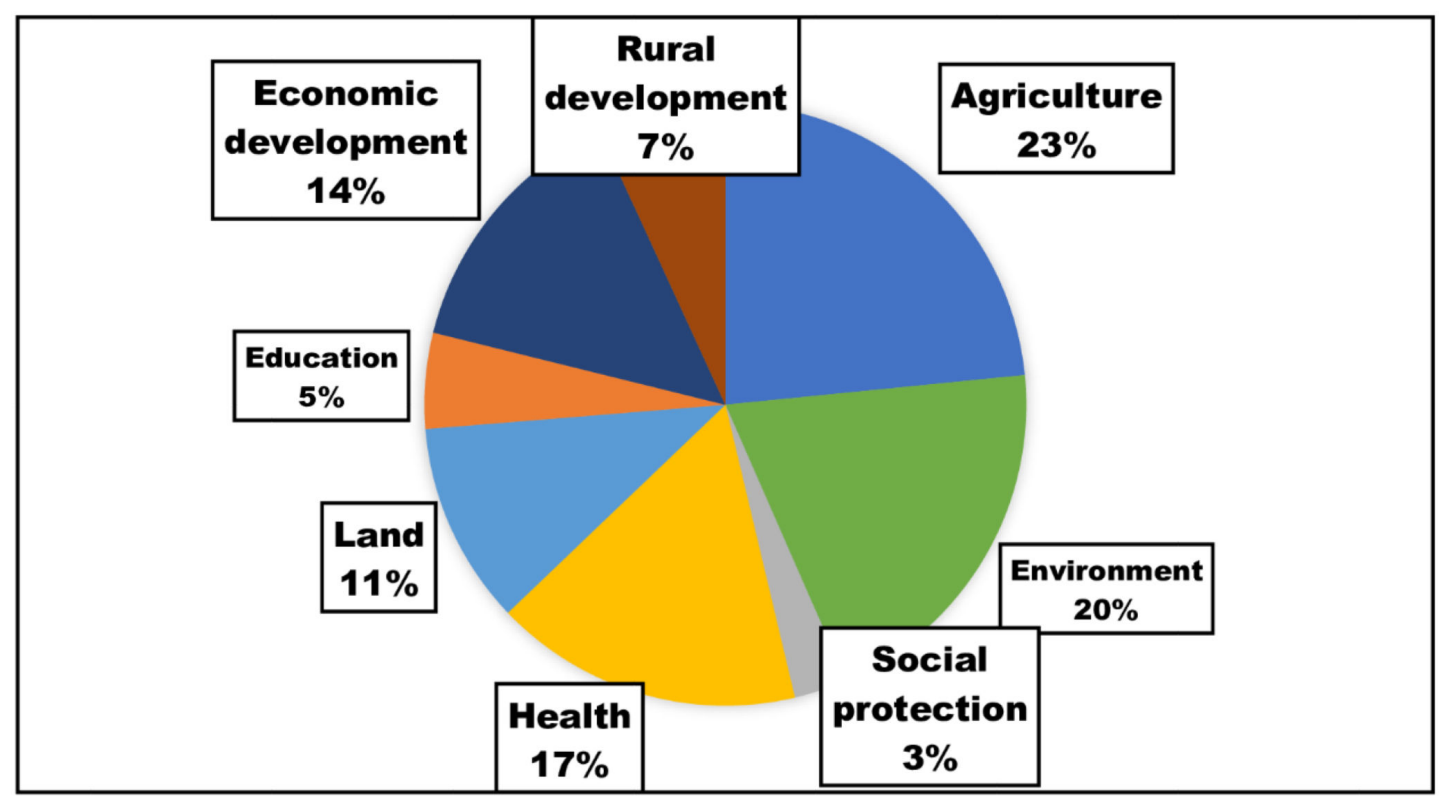
Percentage distribution of policies by the domains of sustainable and healthy food systems

These NPD, MTSF, NGP, NPFNS are “overarching,” with goals across all eight areas. Almost half of the policies focused on agriculture (23%) and environment (20%), with an additional 17%, 14%, and 11% of policies focused on health, economic, and land-related initiatives, respectively (Figure 2). The main sectors covered by the policies generally mirrored the focus areas of the responsible departments. More information is available in Supplementary File 1, where an in-depth description of policies in each domain and specific programmes are described.

### Policy coordination across the sectors

This section provides an analysis of the alignment, coordination, and learning processes that were identified in the selected policies. Overall, there was relative silence in sectoral policies about the involvement of other departments in policy formulation. In contrast, there was a general acknowledgement of the need for other sectors to be involved in policy implementation. However, the mechanisms to achieve this and the ability to learn and adapt during implementation are arguably limited.

### Interdependence of other sectors

We examined interdependence by searching for science, society, and policy actors listed for policy implementation. Under the health domain, The Roadmap for Nutrition in South Africa provides a framework for the department to position nutrition at the centre of the health care system, recognising the multisectoral nature of the challenge and the necessity of coordination and engagement among multiple governmental departments, the private sector, and civil society. The policy identified the Nutrition Directorate at the head office as the unit responsible for the program and listed other government departments, research and tertiary institutions, and development agencies as partners. Specific interventions and activities were allocated to national, provincial and district units. On paper, at least, this reveals a broader multisectoral approach to addressing malnutrition bringing in key actors outside of the state. However, in terms of how to achieve this, the roadmap reverted to generalised statements of intent, including the need for a broad dialogue to provide strategic inputs into social development, agriculture, and rural development (Drimie 2016). Nothing specific or strategically focused was defined.

Under the agricultural domain, the APAP set out to “align itself” with “the New Growth Path, the National Development Plan and Industrial Policy Action Plan*.”* It sought to “assist in the achievement of Outcome 4: Decent Employment through Inclusive Growth and Outcome 7: Comprehensive Rural Development and Food Securit*y*” (DAFF 2015, p. 7). This echoed the APAP predecessor, the IFSS, which listed the “Departments of Health; Social Development; Public Works; Water Affairs and Forestry; Transport; Education; Housing; Provincial and Local Government; Land Affairs; Environment and Tourism; Arts, Culture, Science and Technology” as “core sectors needed for effective implementation (DAFF 2002, p. 7). Once again, the broader multisectoral and multi-stakeholder approach to addressing food security was articulated with lead departments and partners.

Under rural development, the Comprehensive Rural Development Program (CRDP) policy document stated that “inter-departmental collaboration at all spheres of government is essential for the successful implementation of the CRDP,” and that, “projects must be undertaken within a participatory community-based planning approach” (DRDLR 2009, p. 3). However, the Department of Planning, Monitoring and Evaluation (DPME), in their 2013 review of the CRDP, noted that the program has limited progress in uplifting communities, especially creating jobs and community empowerment (Bunce et al. 2013). They also noted that there are also low levels of buy-in, and the will to implement activities at the provincial and local level (Ruhiiga 2017). An evaluation of actual implementation or the translation of these high-level statements into practice was limited, as stated starkly by the DPME, which lay directly under The Presidency.

Under a broader framework that transcends across departments and sectors as the strategy to guide the state, the NDP detailed the need for the interdependence of sectors. Drimie and McLachlan (2013) state that the NDP “provides an innovative framework to begin to inform action required across society to deal with pervasive hunger”, and that the NDP “makes several arguments that resonate with international literature in its appraisal of what it will take to eradicate food insecurity” (p. 218). The NDP necessitates the engagement of entities within the entire food system and numerous linkages throughout multiple sectors and various governmental departments. It can be further argued that NDP policy proposals align with that of a systems approach that subsequently calls for collaboration within the government itself and between the private sector, civil society, and South African citizens.

### Coordination mechanisms

In terms of clearly defined “coordination mechanisms”, the main policies in each domain were assessed for specific institutional arrangements such as the formation of boards/committees, definition of roles and responsibilities of stakeholders. Under the health domain, the DoH was not sufficiently equipped to clearly define mechanisms or institutional arrangements to work in an interdepartmental and multisectoral manner (McLaren et al. 2015), and very little existed to enable coordination with other institutions. In the Roadmap, how the identified partners were to be incorporated was not specified.

Under the agricultural domain, the APAP sets out institutional arrangements that transcend subnational and intersectoral scales. For example, the APAP, hoped to connect farmers with extension officers at the district level, and these in turn with higher-level decision-makers at the national level. The policy states that “the success of APAP lies in our capacity to institutionalise the planning, monitoring, and evaluation thereof. As a consensus document between government, the sector, labour, and civil society, APAP provides a platform of engagement through which the sector and other stakeholders can identify binding constraints and required interventions.” (DAFF 2015, p. 123). However, from experience in the sector, the APAP structure was never effectively implemented, thereby not fulfilling its role as a coordinating mechanism. As stated in the plan and never updated, “this first iteration of APAP is not offered as a fully comprehensive plan; rather, it identifies an ambitious, but manageable number of focused actions, in anticipation of future APAP iterations that will take the process further” (p. 7). No updates on these arrangements have emerged.

Probably the most compelling statement around a coordination mechanism appears in the NPFNS, and the six strategic objectives that anchor the plan. The first objective is to “establish a national multisectoral Food and Nutrition Security Council to oversee alignment of policies, legislation and programmes, and coordination and implementation of programmes and services” (Ngomane 2017, p. 17). This Council would fall under the Deputy President’s office and include DSD; DBE; DTI; DRDLR; Provinces; Local government; Civil Society; Organised Labour and International Development Partners. The immediate task of the Council would include advocating for the integration of policies, legislation, and programmes, to achieve coherence. Further, the 2017 Implementation Plan for the NPFNS, for example, recommended the establishment of (a) an intersectoral National Food and Nutrition Security Council (NFNSC) chaired by the deputy president; (b) Provincial Food and Nutrition Security Councils (PFNSCs) chaired by premiers; (c) district sub-councils on Food and Nutrition Security chaired by mayors; and (d) consultative forums at all levels which are supposed to meet at regular intervals.

Further detail about the Council and the institutional arrangements to underpin it and the other structures are unfortunately missing in any official documentation. Indeed, there has been minimal efforts towards setting these up. The only movement was to establish the National Food and Nutrition Security Coordinating Committee, chaired by the Department of Planning, Monitoring and Evaluation, mainly to steer the implementation of the six strategic objectives as separate entities. This consists of relatively senior officials meeting on an ad hoc basis with some reporting to Parliament with a consistent refrain of having no dedicated budget. Indeed, it is clear from the records of Parliament that the funding required to drive the NPFNS and establish these arrangements, has not been allocated (Parliamentary Monitoring Group 2017). In addition, there have been no clear guidelines or procedures on how the participation of non-state actors, including civil society organisations and the private sector, would be included with regards to the implementation of the policy itself.

### Learning ethos

We searched for indabas^2^, monitoring and evaluation frameworks, and information management as signals of a learning ethos within the policies. The APAP under the agriculture domain placed consensus at its core, stating that “established forums through which all stakeholders are able to interact, table their concerns, and reach consensus with the state around Agriculture, Forestry and Fisheries, on what should be addressed both nationally and provincially” was imperative (DAFF 2015, p. 128). These stakeholders included provincial departments of agriculture, government, sector organisations, labour, and civil society. A striking statement in the IFSS was that “while the strategy takes a long view and is designed to have an enduring impact on food security, it is viewed as a living approach that will be updated as changes comes in the rural economy, national priorities, and external factors” (DAFF 2002, p. 1). One of the key strategies of the IFSS was to establish a Food Insecurity and Vulnerability Information and Mapping System. According to Drimie and Ruysenaar, the system was piloted but never executed (2010). The intention was to create such a learning approach, which, unfortunately, failed in implementation.

The NPFNS detailed a sixth strategic objective as a monitoring and evaluation system for food and nutrition security, including an integrated risk management system for monitoring related risks. The document elaborates that this system would be a national surveillance system that draws on data and metadata from all public and state-owned agencies. And that a set of core indicators for FNS could be integrated into multiple national surveys for continual surveillance. To date, this system remains undeveloped and unfunded.

For the health domain, emphasis was placed on surveys, surveillance, and dialogue to update and monitor the implementation process. The District Health Information System, the Demographic and Health Survey, and the South Africa National Health and Nutritional Examination Survey were among the data sources listed to inform learning. In 2016, the Department of Health conducted the South African Demographic and Health Survey, which contained information on the population’s anthropometric measurements and food security indicators (Department of Health 2019). There was, however, no mention of monitoring and learning within the main environmental, land, and rural development policies. This revealed a data gap, and a lack of systemic learning in this sector.

### Discussion

South Africa has several strategies and plans relating to separate dimensions of the food system. Yet, these have not been effectively translated into programmes with tangible outcomes. The high levels of hunger and malnutrition, including overweight, obesity, and stunting, testify to this (Said-Mohamed et al. 2015; Tathiah et al. 2013). This is essentially a result of implementation challenges and ineffective coordination of these policies, as identified within the NDP. By focusing on the policy component of governance, we have established what policies and which food systems domains they exist. Policy objectives were categorized into the seven policy domains discussed, which then guided the review of 91 policies. About half of the policies reviewed focused on agriculture and the environment, reflecting an emphasis on food availability amongst the food policies. Although more recent policy formulation has broadened the scope away from agriculture to encompass other facets of the food system like nutrition, policy formulations continue to exist in silos. They also offer little tangible mechanisms or solutions to address the inter-linked, systemic issues underpinning food and nutrition insecurity. Of the policies reviewed, six were identified as being “overarching” with goals across all the domains: Nutrition Roadmap, NDP, APAP, CRDP, NPFNS, and the older IFSS. In addition, learning from implementation, and adjusting to improve impact, has been limited.

We observed that agriculture and environment were the dominant local policies in South Africa from 1947 until 2010 when other themes were introduced. This finding is consistent with research conducted in other countries (Lobstein 2007) and can be explained by the impact of international food policies on local agriculture and nutrition agenda (Harris 2019). South Africa’s food security policy is located within a broader regional and international context. Historically, global agriculture and food policies post World War I favored programs on agriculture prosperity, industry development, expansion of the world economy, and rural development (Hawkes et al. 2012). These programs were implemented until the 1970s, when food security was conceptualized as a human right. The human rights perspective brought changes through the Millennium Development Goals and SDGs, which adopt a holistic approach to addressing the root causes of food insecurity (Fanzo 2017). In 2016, the United Nations adopted the 2030 Agenda and its sustainable development goals. Health, poverty reduction, policy coherence, and food security are among the core objectives of the SDGs (Rockström and Sukhdev 2017). Careful consideration of the domains covered by South African policies shows that, even though these policies highlight national development priorities, and many were adopted before the SDGs, they do not deviate from the SDGs. For example, the recognition of nutrition, social protection, and environmental welfare are aligned with the SDGs.

International ideas were localized by influential international stakeholders and multi-national companies active in lobbying, formulating, and implementing food policy. For example, between January 2018 and April 2019, the Department of Agriculture; the Department of Basic Education (DBE); the Department of Health; and the Department of Sport and Recreation had alliances with food processing industries for nutrition education and school nutrition programs (Mialon et al. 2020). Although these alliances are potentially important for the food system, national governments are usually bound by trade and investment commitments that limit their authority and power (Harris 2019; Thow et al. 2018). Secondly, agricultural production could have dominated food policy because it relates to national security issues (International Food Policy Research Institute 2018). It is also often the system’s primary component to be affected by an environmental crisis such as a drought or floods, reinforcing the relationship between the agriculture and environmental sectors (Scott Drimie and Ruysenaar 2010).

### The way forward: towards better coordination

This review assessed main food system domain policies for clearly articulated or defined mechanisms or institutional arrangements to enable coordination (and alignment) amongst different sectors and stakeholders/actors. Most of the policies recognised the need for multisectoral collaboration but did not have structures for carrying out these activities. This highlighted several key challenges to effective policy coordination, namely:

- Siloed approaches: while policies acknowledged the need for other sectors to be involved in policy implementation, there was no effort for cross-sectoral participation during policy implementation.
- No shared understanding: the lack of shared understanding and institutional arrangements to enable coordination. This lack of clarity can limit opportunities for effective coordination at the provincial and district levels.
- Intent lacking action: several progressive policies (e.g., The Roadmap for Nutrition in South Africa 2013-2017) have generalised intent on broad participation during policy formulation. However, they often lacked a strategic roadmap to achieving this; hence the statements of intent never translate into practice.
- Monitoring and evaluation: while the government has developed the Department of Monitoring and Evaluation, an actual evaluation of implementation or translation of these high-level statements of intent into practice is limited.

Although South Africa’s unique socio-political context shapes the challenges listed above, they are not uncommon. Ineffective policy coordination is a global challenge; it has been reported in food systems (Harris 2019; Lang et al. 2009; Parsons and Hawkes 2019), developmental aid (Baker et al. 2018; OECD 2003), and health systems (Assan et al. 2018; Naidoo and Sheiham 2014). Governance structures can explain the lack of coordination (Peters 2018). Firstly, different departments compete for scarce resources and achievement of sectoral targets in a format that prevents cooperation. Secondly, political priorities and mandates hinder collaboration. A review of barriers to nutrition interventions in Africa reported that governments considered ‘visible’ issues such as infrastructure as pressing issues compared to nutrition (Ezezika et al. 2021). Thirdly, lack of clarity on the meaning of coordination, collaboration, roles, and responsibilities of the multisectoral committee (Michaud-Létourneau and Pelletier 2017). In Nepal and Ethiopia, officers involved in a multisectoral nutrition plan were unfamiliar with their roles and requested specific action plans to facilitate implementation (Kennedy et al. 2016).

In moving forward, the NDP provides a useful starting point because it aligns with a systems approach and calls for collaboration within the government, society, and science. However, the question remains - what do successful policies look like, and how can we translate good policy intent into practice and successful outcomes? Mallory and Compton (2019) provide useful case studies of successful policies, and the reader is encouraged to read their book for detailed case studies. We briefly describe how these can be done and provide examples for South Africa:

- There is a need to create cross-sectoral platforms for information, knowledge, and expertise sharing during policy implementation to enable coordination (Peters 2015). The award-winning social protection program of Brazil’s social protection program, the Bolsa Familia used an intersectoral and decentralized structure of governance during implementation to enhance the programs impact (Paiva et al. 2019). This may include pooling resources to achieve cross-sectoral synergies. For example, while the NPFNS is a good policy initiative, it lacks the funding required to drive and establish coordination arrangements.
- Effective coordination should be supported through effective collaboration, i.e., in addition to resource sharing, there should be platforms for joint planning and decision making as was done in the Nordic countries (de La Porte et al. 2020). For example, the NDP provides a useful platform for establishing a shared vision; however, there is a need for Ministerial support for joint planning, decision-making and, evaluation mechanisms. This would empower the Department of Monitoring and Evaluation to monitor policy coordination across various sectors effectively.
- Within these platforms, there is a need to develop clear guidelines or procedures for allowing the participation of non-state actors, including civil society organizations, the private sector, and private citizens. For example, the National Planning Commission (NPC) of South Africa could be revised to allow broader participation. The NPC was established in 2010 to develop a vision and strategic plan for the country while advising the government on cross-cutting issues that influence long-term development. The NPC comprises 24 part-time external commissioners, a chairperson, and a deputy chairperson. Ensuring the participation of all stakeholders’ representatives would contribute to building a shared understanding across a coalition of stakeholders. There needs to be a budget for this to drive the coordination efforts.
- Furthermore, the recognition of nutrition, social protection, and environmental welfare are aligned with the SDGs (Hendriks 2018). This provides an opportunity to follow the ethos of the SDG’s in seeking alignment to achieve sustainable development. It raises a question about what is needed to draw these stated international commitments together to converge in meeting the constitutional commitment to food rights into an overarching food and nutrition security law – a “food goal.” This “food goal” can include transforming food environments, providing climate change mitigation and social security (Kennedy et al. 2021; Ruel and Brouwer 2021). This could begin with an immediate update of the National Policy (NPFNS) to clearly reflect the Sustainable Development Goals and an associated monitoring and evaluation process.

### Conclusions

This review examined national-level policies in South Africa to build evidence on how food is governed through a policy analysis to identify opportunities to improve governance for sustainable and healthy food systems. We identified ninety-one policies that covered eight main domains of the food system: agriculture, environment, economic development, land and land reform, health, education, and social protection. Agricultural production received the most significant emphasis amongst food policies, although more recent formulation has broadened the scope to include livelihoods, social protection, and nutrition. Nonetheless, policy formulations continue to exist in silos offering few tangible mechanisms to address inter-linked, systemic issues.

Important opportunities exist to address these challenges. Recognising the potential for consolidating and reorganising policy to align effectively would build coherence. This should be consolidated by establishing an effective monitoring and evaluation approach that addresses data gaps and encourages opportunities for learning and adapting implementation. Such an approach would help to identify the root causes of the systemic failures and the interconnection of factors that underpin sustainable and healthy food systems. Similarly, actively engaging the existing commitments to the SDGs would help draw these stated international commitments together to meet the constitutional duty to food rights through an overarching food and nutrition security law. Finally, establishing a clear overarching “food goal” within a broader food system framework would help align existing policies, reveal gaps and subsequently reveal the necessary adjustments that need to be implemented to achieve said “food goal”.

## Figures and Tables

**Table 1:** Definition of codes relating to policy coherence and policies selected for each domain for coherence analysis

Codes	Definition
Interdependence of other sectors	Clearly articulated links or connections made with other government sectors in the policy or programme documentation that reveals a more holistic or system-wide approach to food
Coordination mechanisms	Clearly articulated or defined mechanism or institutional arrangement to enable coordination (and alignment) amongst different sectors and stakeholders/ actors in the policy or programme documentation that reveals a more holistic or system-wide approach to food
Learning ethos	Clearly articulated process of learning (reflection on what is emerging and adaptation if necessary) defined in policy or programme documentation that reveals the need for a learning approach or ethos rather than a prescription of solutions
**Food system domain**	**Policy(ies) selected for coherence analysis**
Agriculture	Agricultural Policy Action Plan 2015-2019, National Food and Nutrition Security Plan
Environment	National Environmental Management Act
Economic development	National Development Plan
Health	Roadmap for Nutrition in South Africa 2013-2017
Education	National School Nutrition Programme
Land reform	Policy was at draft stage at the time of study
Rural development	Comprehensive Rural Development Program (CRDP)
Social protection	Social Assistance Act
Overarching	National Development Plan

Source: Authors

## Data Availability

All data on which this manuscript is based are in Supplementary File 1.

## Declarations

1 USSD were calculated on 24/02/2020 using Oanda currency converter

2 An indaba is a conference or seminar with stakeholders
